# Evolutionary dynamics and functional diversification of BBX transcription factors in C_4_ grasses from *Setaria italica* and *Setaria viridis*

**DOI:** 10.3389/fpls.2025.1701242

**Published:** 2026-01-22

**Authors:** Jiaxin Liu, Haoshan Zhang, Ling Zhao, Hua Wang, Shen Li, Yulu Wang, Lin Li, Luming Zou, Ting Zhang, Ruhong Cheng, Zhigang Shi, Zhuxin Zhang, Yujie Du, Yuhan Sun, Hui Gao, Genping Wang

**Affiliations:** 1 Hebei Key Laboratory of Crop Stress Biology, Department of Life Science and Technology, College of Marine Resources and Environment, Institute of Wild Plant Resources Application, Hebei Normal University of Science and Technology, Qinhuangdao, China; 2 Institute of Millet Crops, Hebei Academy of Agriculture and Forestry Sciences/Key Laboratory of Genetic Improvement and Utilization for Featured Coarse Cereals (Co-construction by Ministry and Province), Ministry of Agriculture and Rural Affairs/National Foxtail Millet Improvement Center/Key Laboratory of Minor Cereal Crops of Hebei Province, Hebei Academy of Agriculture and Forestry Sciences, Shijiazhuang, China

**Keywords:** BBX, C4 crops, evolutionary dynamics, functional diversification, *Setaria italica*

## Abstract

**Introduction:**

B-box (BBX) transcription factors are key regulators of plant development, growth, and responses to photoperiod. However, their evolutionary dynamics and functional diversification in C_4_ grass crops are limited.

**Methods and results:**

The study involved the identification and systematic analysis of 33 *BBX* genes from *Setaria italica* (16) and *Setaria viridis* (17), which were classified into subfamilies I, III, and IV based on phylogenetic relationships. Gene structure and motif analysis revealed conserved patterns within subfamilies, while chromosomal mapping and duplication analysis suggested that dispersed duplication was the primary driver of *BBX* gene family expansion, with all genes under purifying selection. Comparative genomic analyses across representative species of the Poaceae and *Arabidopsis thaliana* indicated a contraction of the *BBX* gene family in C_4_ grasses. Expression profiling suggests potential functional divergence, with *BBX* genes exhibiting differential expression patterns associated with development, photoperiod response, and abiotic stress. *Cis*-acting element analysis further highlighted species-specific regulatory mechanisms. Several *SiBBX* genes showed clear daily rhythmic expression under long-day photoperiod conditions by quantitative real-time PCR (qRT-PCR). Subcellular localization assays showed that selected SiBBX proteins localize to the nucleus, consistent with their roles as transcription factors.

**Discussion:**

Our findings provide insights into the molecular evolution and functional diversification of *BBX* genes in C_4_ grasses and offer potential targets for genetic improvement of heading date and stress tolerance in C_4_ crops.

## 1 Introduction

Light affects seed germination, geotropism, seedling yellowing, circadian rhythm, and flowering time in plants (Mehta et al., 2023; Wei et al., 2023; Wang et al., 2024a). The zinc finger transcription factors known as BBX (B-box) proteins have B-box domains and are extensively involved in light-regulated plant functions, including hormone response, photomorphogenesis, flowering time regulation, light signal transduction, and abiotic stress adaptation (Khanna et al., 2009; Gangappa and Botto, 2014).

At the N-terminus, BBX proteins contain 1–2 conserved B-box domains. Among some BBX proteins, CCT (CONSTANS, CO-like, and TOC1) domain is present at the C-terminus (Khanna et al., 2009), which is crucial flowering regulators. In *Arabidopsis thaliana*, CO/*AtBBX1* acts as a key role of flowering under long-day photoperiod by CCT domain binding to the promoter of FT (FLOWERING LOCUS T) to promote flowering (Tiwari et al., 2010). Other CO-LIKE (COL) proteins also regulate flowering. For example, *AtBBX4* and *AtBBX7* act as flowering inhibitors (Cheng and Wang, 2005; Datta et al., 2005), while *AtBBX6* promotes flowering in synergy with other regulatory factors (Hassidim et al., 2009). In rice, *OsBBX2* interacts with *Hd1* (*OsBBX15*) to synergistically inhibit the transcription of *Hd3a*, delayed flowering (Yang et al., 2024). In both long-day and short-day photoperiods, *OsBBX14* inhibits the expression of *Hd3a* and *RFT1*, delayed flowering (Bai et al., 2016).

Apart from BBXs containing the CCT domain, flowering is negatively regulated by *AtBBX32* in a dose-dependent manner (Park et al., 2011). In addition, BBX proteins are involved in circadian rhythm. AtBBX19 interacts with PRRs to regulate circadian rhythm (Yuan et al., 2021). *AtBBX20*, *AtBBX21*, and *AtBBX22* are essential co-factors in HY5-dependent regulation that are involved in transcriptional regulation, anthocyanin accumulation, and hypocotyl elongation (Bursch et al., 2020).

Additionally, hormone-regulated plant development and abiotic stress responses are mediated by BBX proteins. *AtBBX16* regulates auxin synthesis by controlling *SUR2*, thereby regulating branch development (Zhang et al., 2014). *AtBBX18* regulates hypocotyl elongation by controlling GA synthetic and metabolic genes (Wang et al., 2010). HY5 directly targets AtBBX7 and AtBBX8, which modulate the expression of several cold-responsive genes to positively regulate cold tolerance (Li et al., 2021). AtBBX18 and AtBBX23 interact with ELF3 to regulate thermomorphogenesis (Ding et al., 2018). CmBBX22 regulates abscisic acid (ABA) signaling and controls the drought tolerance in chrysanthemum (Liu et al., 2022).

Although *BBX* functions have been intensively studied in C_3_ species, their evolutionary patterns, duplication history, regulatory diversity, and expression behavior in typical C_4_ grasses remain largely unresolved. Compared to C_3_ plants, C_4_ grasses exhibit distinct photoperiod sensitivity, photosynthetic efficiency, and environmental adaptability (Guidi et al., 2019; Pardo and VanBuren, 2021; Li et al., 2023). However, it is still unclear whether *BBX* gene family evolution in C_4_ lineages follows the same rules as in C_3_ species, which *BBX* members underwent C_4_-specific duplication or functional diversification, and how *BBX* genes contribute to C_4_-specific light signaling or stress adaptation. These unresolved questions represent a major knowledge gap in understanding *BBX* regulatory evolution in grasses.

Foxtail millet (*Setaria italica*) is an important dryland cereal crop originating from the Yellow River basin in China, characterized by drought tolerance, poor soils tolerance, a short growth period, and high nutritional value (Lee et al., 2007; Doust et al., 2009). Green millet (*Setaria viridis*) is considered the domesticated ancestor of foxtail millet, and both belong to *Setaria* within the Poaceae family, representing typical C_4_ photosynthetic plants (Fukunaga and Kawase, 2024). Due to the small size of their genomes, short life cycles, relatively simple genetic operations, and the completed whole-genome sequencing and annotation, foxtail millet and green millet are commonly used as model systems for investigating C_4_ photoperiod regulation and stress adaptation (Lata et al., 2012; Zhang et al., 2012; Yang et al., 2020). In recent years, genome-wide identification and analysis provide an efficient strategy for predicting the functions of genes in the gene family. Extensive research has been conducted on the *BBX* gene family in various C_3_ plant species, including *Arabidopsis thaliana* (Gangappa and Botto, 2014)*, Nicotiana tabacum* (Song et al., 2022)*, Oryza sativa (*Shalmani et al., 2019*), Glycine max* (Shan et al., 2022)*, Fagopyrum tataricum* (Zhao et al., 2021)*, Medicago* (Wang et al., 2024b)*, Trichosanthes kirilowii* (Li et al., 2025), yielding valuable insights into BBX function in C_3_ systems. However, systematic analyses in C_4_ grasses are still missing, especially regarding whether C_4_ evolution shaped *BBX* gene expansion, synteny conservation, promoter architecture, or photoperiod-responsive expression patterns.

To address these gaps, we performed a genome-wide characterization of the *BBX* gene family in the C_4_ model *Setaria*. Comprehensive analyses of protein properties, conserved motifs, gene structure, promoter *cis*-elements, molecular evolution, and expression profiles revealed candidate *SiBBX* members that may play central roles in light response and environmental stress. This study provides the comprehensive overview of *BBX* evolution in *Setaria* and identifies candidate *SiBBX* genes potentially involved in light signaling and stress responses in C_4_ grasses. These findings establish a molecular framework for understanding BBX functional evolution in C_4_ systems and offer promising targets for improving heading date and stress tolerance in C_4_ crops.

## 2 Materials and methods

### 2.1 Identification of *BBX* Genes in *Setaria italica* and *Setaria viridis*

To identify *BBX* gene family members, we employed a combined approach of HMM-based domain search and BLASTP similarity search. First, the Hidden Markov Model (HMM) profile of the B-box domain (Pfam accession: PF00643) was acquired from the Pfam database (http://pfam.xfam.org/) (Finn et al., 2013). HMMER v3.0 was used to perform a search against the annotated protein sequences of *Arabidopsis thaliana*, *Oryza sativa*, *Triticum aestivum*, *Zea mays*, *Sorghum bicolor*, *Setaria viridi*s and *Setaria italica* (downloaded from Phytozome database: https://phytozome-next.jgi.doe.gov/) with an E-value cutoff of 1e-5. In parallel, protein BLAST (BLASTP) was performed using known *Arabidopsis thaliana* BBX protein sequences as queries to search against the *Oryza sativa*, *Triticum aestivum*, *Zea mays*, *Sorghum bicolor*, *Setaria viridi*s and *Setaria italica* protein databases. To improve specificity, only hits with a sequence identity of >30% were retained. These thresholds (E-value < 1e-5 and identity > 30%) have been widely applied in transcription factor family identification to ensure both sensitivity and specificity and to avoid spurious matches (Fang et al., 2025). Across the two species, HMMER initially identified 16 candidates in *Setaria italica* and 17 in *Setaria viridi*s, whereas BLASTP identified 52 and 50, respectively. Candidates detected by both HMMER and BLASTP were treated as high-confidence BBX proteins. All putative BBX proteins were then confirmed by domain annotation using SMART (http://smart.embl-heidelberg.de/) (Schultz et al., 1998) and NCBI’s Conserved Domain Database (CDD) (Marchler-Bauer et al., 2014). Proteins lacking at least one conserved B-box domain were excluded from subsequent analyses. After removing sequences lacking a complete B-box domain based on SMART and CDD verification, a final set of 33 *BBX* genes (16 in *Setaria italica* and 17 in *Setaria viridis*) was retained for downstream analyses. Using the online tool ProtParam (http://web.expasy.org/protparam/), the typical characters (the theoretical isoelectric point (PI), the instability index, the aliphatic index, and the molecular weight (MW)) were examined. WoLF PSORT, an online tool (https://wolfpsort.hgc.jp), was used to predict the subcellular localization of proteins.

### 2.2 Phylogenetic tree and gene structure characterization analysis

Multiple sequence alignment of BBX protein sequences from the corresponding species datasets was performed using MAFFT v7 with default gap-handling parameters (Katoh, 2002). Phylogenetic trees were inferred using IQ-TREE v2.1.2 under the Maximum Likelihood framework (Nguyen et al., 2014). Phylogenetic relationships were inferred with IQ-TREE v2.1.2 under the Maximum Likelihood framework. For both the three species (*Arabidopsis thaliana, Setaria viridi*s and *Setaria italica*) and seven species datasets (*Arabidopsis thaliana*, *Oryza sativa*, *Triticum aestivum*, *Zea mays*, *Sorghum bicolor*, *Setaria viridi*s and *Setaria italica*), ModelFinder was used to select the best substitution model. Based on the Bayesian Information Criterion (BIC), the Blosum62+F+R5 model (three species) and VT+F+R7 model (seven species). Ultrafast bootstrap approximation was used to assess branch support. Final trees were visualized with iTOL (https://itol.embl.de/). Gene structure information (exon-intron organization) was obtained from GFF3 annotation files and visualized using TBtools (Chen et al., 2020). MEME Suite was used to find conserved motifs, with a maximum of 10 motifs and default settings.

### 2.3 Chromosomal localization and gene duplication analysis

MapChart was used to visualize the *BBX* genes’ chromosomal locations, which were mapped using the genome annotation files (Voorrips, 2002). MCScanX was employed to identify gene duplication events and classify them into five types: singleton, dispersed, proximal, tandem, and segmental duplications (Wang et al., 2012). Syntenic relationships between *Setaria italica* and six other species (*Arabidopsis thaliana*, *Oryza sativa*, *Triticum aestivum*, *Zea mays*, *Sorghum bicolor*, and *Setaria viridi*s) were also identified using MCScanX and visualized with Circos plots (Krzywinski et al., 2009; Wang et al., 2012). Synonymous (Ks) and nonsynonymous (Ka) substitution rates were computed using the KaKs Calculator to evaluate the pressure of evolutionary selection (Zhang, 2022). The following formula was utilized to estimate the divergence time (T) between orthologous gene pair: T=Ks/2r (Huang et al., 2015). For monocots, the neutral substitution rate is 6.5 × 10–^9^ substitutions per site annually (Gaut et al., 1996). Ks values exceeding 2.0 or equal to 0 were excluded to avoid unreliable estimates. The resulting T values were used to infer the divergence times of *BBX* orthologs across species.

### 2.4 *BBX* gene gain-loss dynamics analysis

Gene family expansion and contraction of the *BBX* family were analyzed using CAFE5 (Mendes et al., 2020), which models gene copy number evolution under a stochastic birth-death process. The ultrametric species tree used for the analysis was constructed based on published divergence times, and *BBX* copy numbers for *Arabidopsis thaliana, Oryza sativa, Triticum aestivum, Zea mays, Sorghum bicolor, Setaria italica* and *Setaria viridis* were used as input. CAFE was run under a single–λ model, and the estimated rate parameter (λ = 0.0111) indicated a suitable fit for the dataset. Ancestral *BBX* copy numbers and branch-specific gain-loss events were inferred using CAFE’s maximum-likelihood framework, with *p*-values calculated (significant expansion or contraction was determined at *p* < 0.05 (De Bie et al., 2006). The probability of gene gain or loss along each branch was obtained from CAFE’s posterior probability output and summarized in **Supplementary Table 4**.

### 2.5 RNA-seq expression analysis

**Figure 1:**
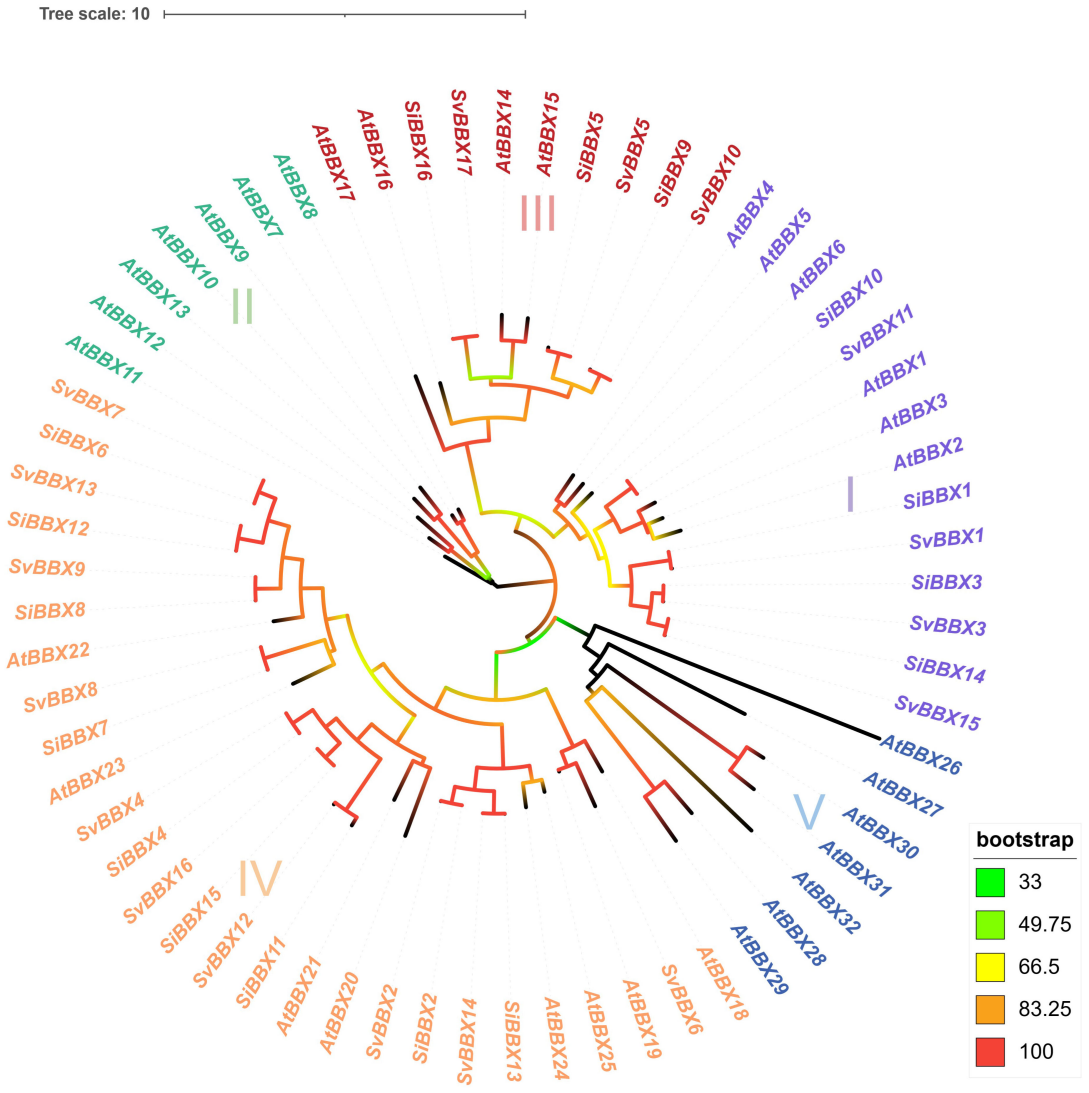
Phylogenetic trees illustrating the analysis of BBX protein sequences from *Setaria italica, Setaria viridis* and *Arabidopsis thaliana*. The phylogenetic tree was constructed using the Maximum Likelihood (ML) method in IQ-TREE. This tree delineates five distinct phylogenetic subfamilies. Each subfamily is represented by a differently colored square: purple (I), green (II), red (III), yellow (IV) and blue (V). Branch colors represent bootstrap support values visualized using iTOL's color-gradient mode, where red indicates high support (bootstrap=100) and green indicates lower support (bootstrap=33). Only bootstrap values ≥33 are displayed.

To examine the transcriptional patterns of *BBX* genes in *Setaria italica* and *Setaria viridis*, we retrieved publicly available RNA-seq expression data from the Phytozome GeneAtlas v2 database (https://phytozome-next.jgi.doe.gov/). GeneAtlas v2 provides a fully pre-processed and standardized expression matrix quantified as fragments per kilobase per million mapped reads (FPKM), generated using a unified pipeline across all tissues and conditions. Therefore, no additional read trimming, alignment, or re-normalization steps were applied in this study. Since the GeneAtlas dataset does not include replicate-level variance information, no statistical differential expression testing was performed. Instead, *BBX* expression patterns were assessed by comparing relative FPKM abundance across developmental stages and stress-related treatments. For visualization purposes, expression values were transformed using Log_10_(FPKM) and displayed as a heatmap generated with TBtools (Chen et al., 2020). Because this dataset does not include biological replicates, the heatmap in **Figure 1** reflects qualitative expression patterns rather than statistical comparisons.

### 2.6 Promoter *Cis*-acting element identification and enrichment analysis

To identify stress-, hormone-, and light-responsive *cis-*acting elements, the PlantCARE database (http://bioinformatics.psb.ugent.be/webtools/plantcare/) was used to analyze the 2,000 bp upstream sequences of *BBX* genes that were extracted as putative promoter regions (Lescot, 2002).

To determine whether specific *cis*-acting elements were non-randomly enriched in *BBX* promoters relative to the genomic background, we performed a *cis-*acting element enrichment test using Fisher’s exact test. Enrichment significance was assessed using two-tailed Fisher’s exact tests, and *p*-values were corrected for multiple testing using the Benjamini–Hochberg false discovery rate (FDR) in **Supplementary Table 8**. Motifs with FDR < 0.05 were considered significantly enriched or depleted. All statistical analyses were conducted in R (version 4.3.0).

### 2.7 Plant growth conditions

Foxtail millet (*Setaria italica* cv. Longgu 26) seedlings were cultivated in regulated growth chambers with a long-day photoperiod (15 hours of light and 9 hours of darkness), 28°C/22°C day/night temperature, and 60% relative humidity. For daily rhythm expression analysis, leaf samples were collected at six time points (9:00, 13:00, 17:00, 21:00, 01:00, and 05:00; light-period from 9:00 to 24:00 and dark-period from 24:00 to 9:00). For extracting RNA, samples were kept at -80 °C and frozen in liquid nitrogen.

### 2.8 RNA isolation and qRT-PCR examination

The Fastpure Universal Plant Total RNA Isolation Kit (Vazyme China RC411-01) was used to extract total RNA, and the PrimeScript RT reagent kit (Vazyme China RT101-01) was used to create first-strand cDNA. Using a LightCycler 480 system (BioRAD CFX96 Optics Module), qRT-PCR was carried out using SYBR Green Master Mix (Vazyme China Q711-02). The cullin gene of *Setaria italica* (*Seita.3G037700*) was used as an internal reference. We used the 2^^−ΔΔCt^ method to calculate relative expression levels (Livak and Schmittgen, 2001). For every sample, three technical and three biological replicates were carried out. A list of the qRT-PCR primers is provided in **Supplementary Table 9**.

### 2.9 Subcellular localization

The leaves of *Nicotiana benthamiana* were used to temporarily express the coding sequences of specific *SiBBX3*, *SiBBX5*, *SiBBX8*, and *SiBBX13* genes that had been cloned into *pCAMBIA1300-35s-GFP* vectors. A Leica TCS SP8 confocal laser scanning microscope was used to view the GFP fluorescence.

## 3 Result

### 3.1 *BBX* identification and structural characterization in *Setaria italica* and *Setaria viridis*

To identify *BBX* gene family members, we combined an HMM-based domain search with BLASTP similarity searches. The B-box domain in candidate proteins was further validated using the online tools SMART and CDD. In the *Setaria*, 33 *BBX* genes were found, including 16 in *Setaria italica* and 17 in *Setaria viridis*, These genes were named *SiBBX1*–*SiBBX16* (*Setaria italica*) and *SvBBX1*–*SvBBX17* (*Setaria viridis*) based on their chromosomal localization order (**Supplementary Table 1**). In addition, the members of *BBX* family in *Setaria* have predicted molecular weights between 22.2 and 50.2 kDa, and their protein lengths range from 148 amino acids (SvBBX16) to 503 amino acids (SvBBX10). The theoretical isoelectric point (pI) ranges from 4.79 to 6.65, and the instability index indicates that most BBX proteins have moderate stability (index < 60) (**Supplementary Table 1**). All BBX proteins exhibit negative Grand average of hydropathicity (GRAVY) scores, suggesting that they are intrinsically hydrophilic (**Supplementary Table 1**).

To provide an overview of the evolutionary relationships of *BBX* genes in *Setaria*, we first constructed a phylogenetic tree. Using the model plant *Arabidopsis thaliana* as a reference, the *BBX* gene family members were systematically classified. According to the results, the *BBX* genes were divided into only three subfamilies, whereas the *BBX* genes in *Arabidopsis thaliana* were classified into five subfamilies (I-V). In *Setaria italica*, subfamily IV has the highest number of members (9 members), followed by subfamily I (4 genes) and subfamily III (3 genes) (**Figure 1**, **Supplementary Table 1**). A similar distribution was observed in *Setaria viridis* (**Figure 1**, **Supplementary Table 1**).

To investigate the evolutionary conservation of BBX proteins in foxtail millet and green millet, we analyzed the conserved motifs of 65 *BBX* gene family members in *Setaria italica*, *Setaria viridis*, and *Arabidopsis thaliana*s. The analysis revealed that 10 conserved motifs were found and the distribution pattern of motifs in BBX proteins belonging to the same subfamily was similar. All *BBX* genes contain at least one Motif 1 and exhibit high conservation in three species, with the highest number of motifs in subfamily III and the lowest in subfamily V (**Supplementary Figure 1**, **Supplementary Table 1**). Additionally, Motif 2 and Motif 8 also show significant conservation in BBX proteins of subfamily III (**Supplementary Figure 1**, **Supplementary Table 1**).

The structural patterns of *BBX* genes were examined to investigate the diversity of *BBX* genes in foxtail millet and green millet. The results showed that most *BBX* genes had 1–3 introns and 1–2 exons. Among these, the structural patterns of the III subfamily genes in foxtail millet and green millet were highly conserved, consisting of 2 exons and 3 introns (except for *SvBBX5*) (**Supplementary Figure 1**, **Supplementary Table 1**). According to subcellular localization predictions, the majority of BBX proteins are found in the nucleus, which is in line with their role as transcription factors. The predicted localization of certain BBX proteins to chloroplasts or the cytoplasm, implying potential non-transcriptional or multi-compartmental roles (**Supplementary Table 1**).

### 3.2 Chromosome distribution and *BBX* gene duplication analysis in *Setaria italica* and *Setaria viridis*

**Figure 2:**
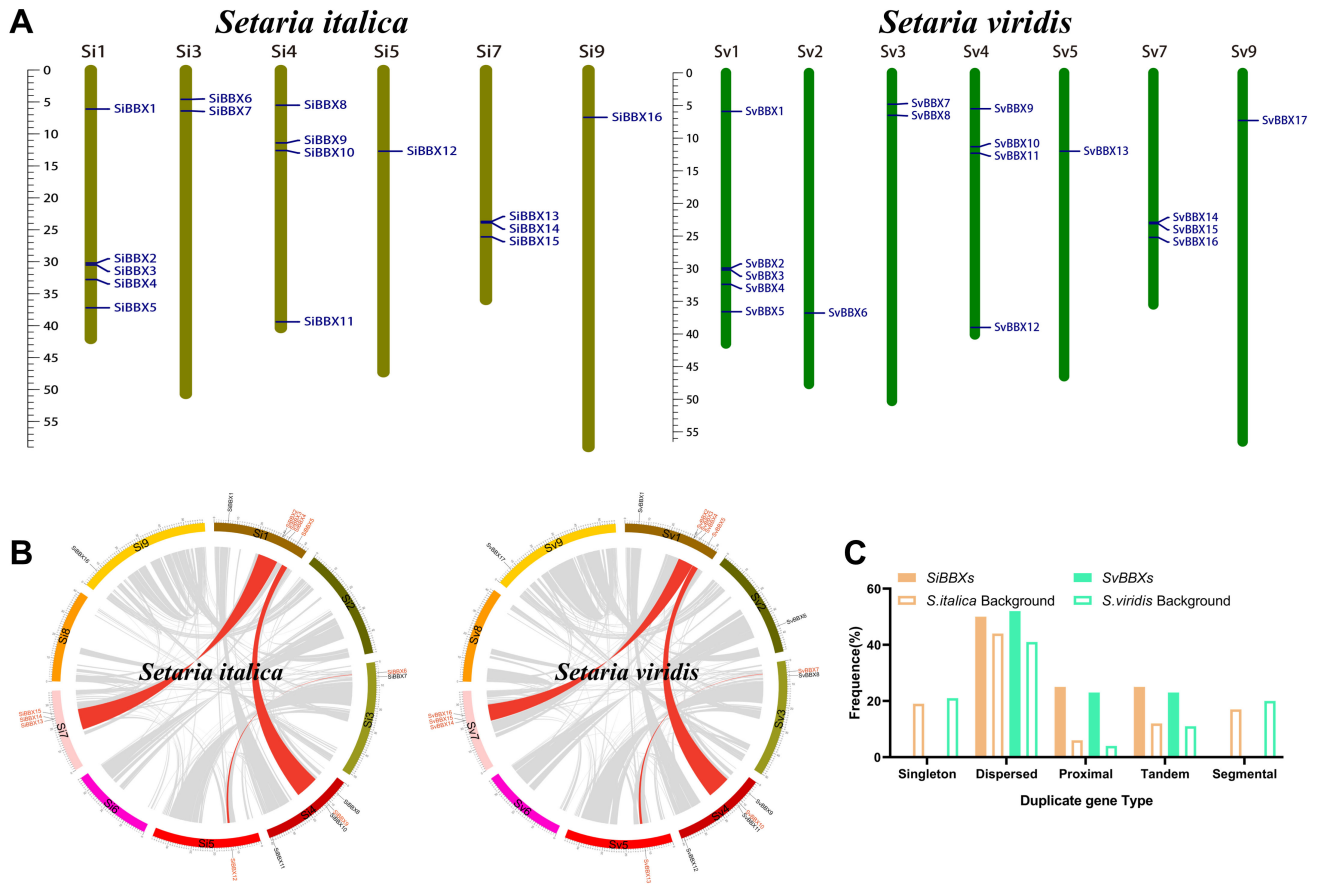
Schematic Representation of Chromosomal Distribution and Interchromosomal Relationships of *BBX* Genes in Setaria. **(A)** Karyotype localization of *BBX* genes on the chromosomes of foxtail millet (*Setaria italica*) and green millet (*Setaria viridis*). **(B)** Synteny analysis of *BBX* genes within the foxtail millet (*Setaria italica*) and green millet (*Setaria viridis*) genome. Gray lines represent all syntenic genes, while red lines indicate synteny relationships between *BBX* genes. **(C)** The different types of BBX gene duplications—singleton, dispersed, proximal, tandem, and segmental—were quantified in foxtail millet (*Setaria italica*) and green millet (*Setaria viridis*). Open boxes represent the whole-genome level.

The *BBX* gene family members in foxtail millet and green millet were mapped onto chromosomes to reveal their genomic structure and distribution patterns. Based on the genomic maps of *BBX* members in foxtail millet and green millet, we found that *SiBBXs* are distributed across six chromosomes, while *SvBBXs* are distributed across seven chromosomes. Notably, *BBX* genes in *Setaria* are unevenly distributed across the chromosomes. The most *BBX* genes are located on Chr.1, whereas Chr.5 in both foxtail millet and green millet has only one *BBX* gene. One *BBX* gene (*SvBBX*6) was found on Chr.2 in green millet, whereas none were present in foxtail millet (**Figure 2A**, **Supplementary Table 1**).

The duplication events of the *BBX* gene in foxtail millet and green millet were analyzed by MCScanX. Five pairs of segmental duplication genes were detected in *Setaria italica* and *Setaria viridis*, respectively (**Figure 2B**; **Supplementary Table 2**). Duplicated genes were mainly located on different chromosomes, indicating that the expansion of the *BBX* gene family was primarily driven by segmental duplication rather than tandem duplication. The analysis of the duplication types of *BBX* family members revealed that among 16 *SiBBXs* and 17 *SvBBXs*, 50% belonged to dispersed duplication, with proximal and tandem duplication each accounting for approximately 25% (**Figure 2C**, **Supplementary Table 3**).

The evolutionary dynamics of the duplicated genes were evaluated by calculating non-synonymous (Ka), synonymous (Ks) and Ka/Ks ratios. As all Ka/Ks values were < 1, the genes appear to have undergone purifying selection, reflecting functional conservation (**Supplementary Table 2**).

### 3.3 Evolutionary relationships and gain-loss dynamics of *BBX* gene in *Setaria* and other species

**Figure 3:**
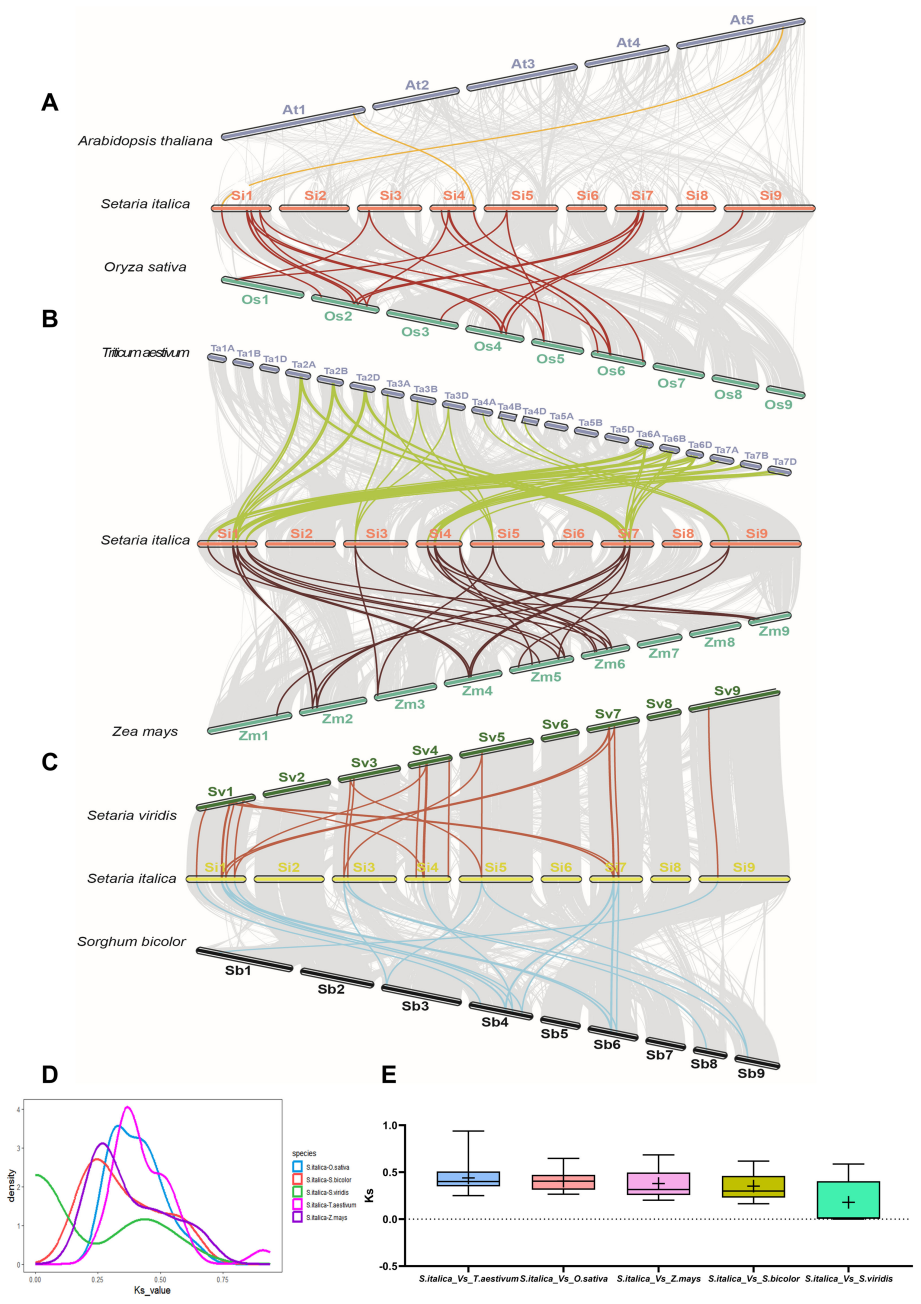
Synteny analysis of *BBX* genes between foxtail millet, and other six plant species. **(A)** Synteny analysis of the *BBX* genes between *Arabidopsis thaliana, Oryza sativa*, and *Setaria italica*. **(B)** Synteny analysis of the *BBX* genes between *Triticum aestivum, Zea mays*, and *Setaria italica*. **(C)** Synteny analysis of the *BBX* genes between *Setaria viridis, Sorghum bicolor*, and *Setaria italica*. **(D)** The distribution of Ks values for *BBX* genes between *Setaria italica* and *Setaria viridis, Triticum aestivum, Zea mays, Oryza sativa*, and *Sorghum bicolor*. **(E)** Box plot of Ks values for *BBX* genes between *Setaria italica* and *Setaria viridis, Triticum aestivum, Zea mays, Oryza sativa*, and *Sorghum bicolor*.

To investigate the evolution of the *BBX* gene family in C_4_ plants of *Setaria*, we used *Setaria italica* as the core species to construct a synteny map between *Setaria italica* and six representative species (*Arabidopsis thaliana*, *Oryza sativa*, *Triticum aestivum*, *Zea mays*, *Sorghum bicolor* and *Setaria viridi*s). The results showed that 16 *SiBBXs* were significantly syntenic with 16 genes in *Setaria viridi*s. In other species, *SiBBXs* were syntenic with 16 genes in *Sorghum bicolor*, 22 genes in *Zea mays*, 41 genes in *Triticum aestivum*, 16 genes in *Oryza sativa*, and 2 genes in *Arabidopsis thaliana*. Further analysis revealed that foxtail millet formed 26, 67, 26, 36, 26, and 2 pairs of orthologous gene pairs with green millet, wheat, sorghum, maize, rice, and Arabidopsis, respectively (**Figures 3A-C**, **Supplementary Table 2**).

Selection pressure analysis revealed that the Ka/Ks ratios of all orthologous genes < 1, indicating that these genes were under purifying selection (**Supplementary Table 2**). The Ks distribution and synteny analysis of orthologous *BBX* genes between *Setaria italica* and other species showed a major differentiation peak (**Figures 3D, E**, **Supplementary Table 2**). Based on the average synonymous substitution rate of 6.5 × 10^-9^ for Poaceae (Gaut et al., 1996), the divergence times for *BBX* genes can be estimated as follows: approximately 26.9 million years for foxtail millet and rice/wheat, approximately 19.2 million years for foxtail millet and maize/sorghum, and approximately 0.8 million years for foxtail millet and green millet (**Supplementary Table 2**).

In addition, *BBX* gene gain-loss dynamics analysis showed that approximately 90 million years ago, the common ancestor of seven species (*Arabidopsis thaliana*, *Oryza sativa*, *Triticum aestivum*, *Zea mays*, *Sorghum bicolor* and *Setaria viridi*s and *Setaria italica*) had at least 28 *BBXs*. Subsequently, the number of *BBXs* expanded to 32 in *Arabidopsis thaliana*, while the common ancestor of the six grass plant (*Oryza sativa*, *Triticum aestivum*, *Zea mays*, *Sorghum bicolor*, *Setaria viridi*s and *Setaria italica*) possessed only 26 genes. The number of genes then gradually decreased, with the common ancestor of the four C_4_ plants in the Panicoideae (*Sorghum bicolor*, *Zea mays,Setaria viridi*s and *Setaria italica*), which having only 23 genes. Furthermore, in maize and sorghum, the number of *BBXs* in maize showed an increasing trend, while the number of *BBXs* in sorghum continued to decrease; the number of *BBXs* in *Setaria* species (foxtail millet and green millet) decreased to 16 and 17 (**Supplementary Figure 2**). Although branch-wise probability support varied, the overall contraction was statistically significant at the family level (*p* = 0.002) (**Supplementary Table 4**). This result indicates that the *BBX* gene family of C_4_ grasses (foxtail millet, green millet, and sorghum) tended to shrink.

**Figure 4:**
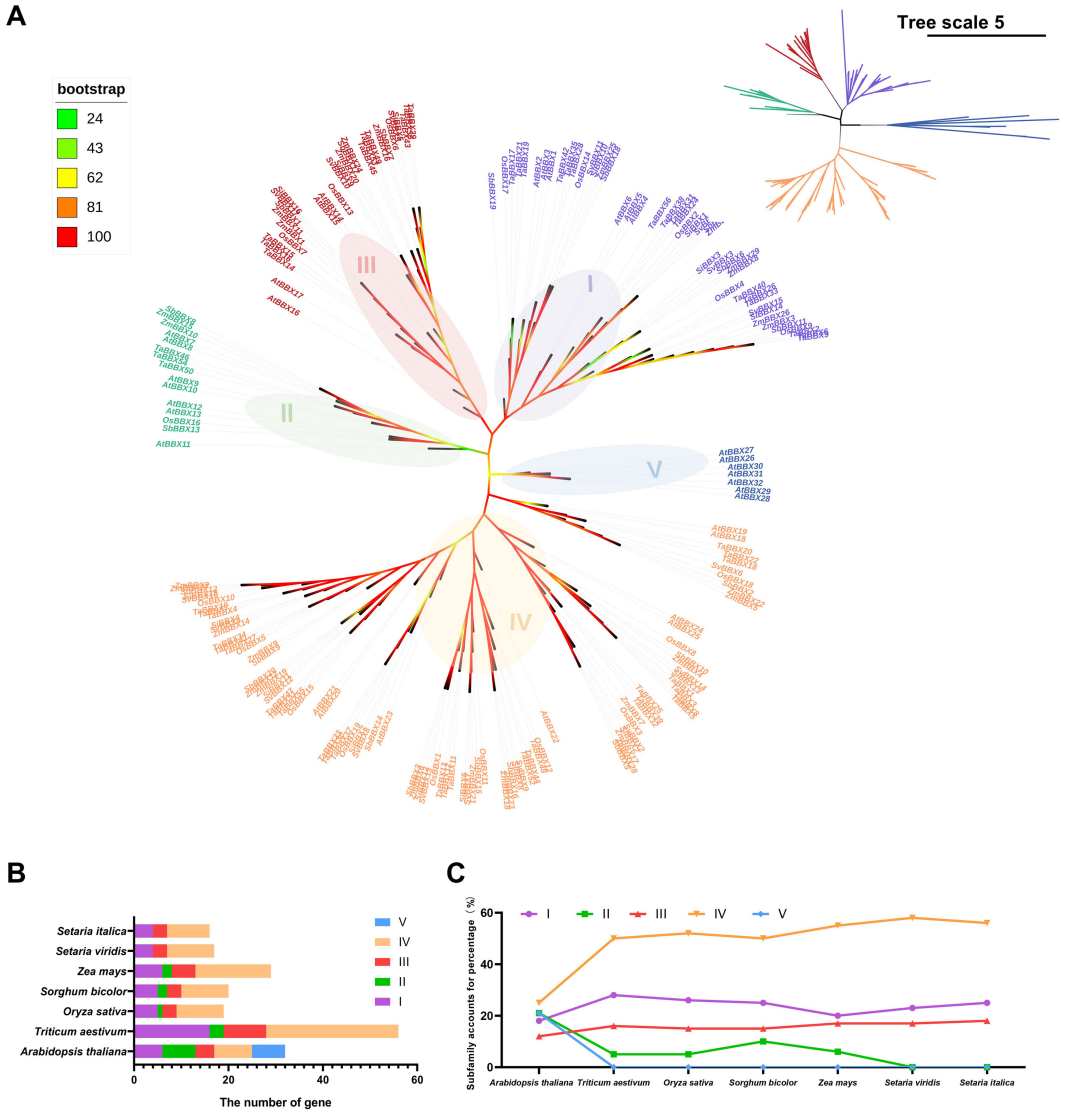
Phylogenetic trees illustrating the analysis of BBX protein sequences from *Setaria italica*, *Setaria viridis*, *Triticum aestivum*, *Zea mays*, *Oryza sativa*, *Sorghum bicolor* and *Arabidopsis thaliana*. **(A)** The phylogenetic tree was constructed using the Maximum Likelihood (ML) method in IQ-TREE. This tree delineates five distinct phylogenetic subfamilies. Each subfamily is represented by a differently colored square: purple (I), green (II), red (III), yellow (IV) and blue (V). Branch colors represent bootstrap support values visualized using iTOL's color-gradient mode, where red indicates high support (bootstrap=100) and green indicates lower support (bootstrap=33). Only bootstrap values $\ge$33 are displayed. **(B)** Statistics on the number of different subfamilies of BBX genes in *Setaria italica*, *Setaria viridis*, *Triticum aestivum*, *mays*, *sativa*, *Sorghum bicolor* and *Arabidopsis thaliana*. **(C)** A comparative analysis of the proportions of different BBX gene subfamilies in *Setaria italica*, *Setaria viridis*, *Triticum aestivum*, *Zea mays*, *Oryza sativa*, *Sorghum bicolor*, and *Arabidopsis thaliana*.

To investigate the retention of *BBX* gene family members during species evolution, we constructed a phylogenetic tree using 189 BBX protein sequences from *Arabidopsis thaliana*, *Oryza sativa*, *Triticum aestivum*, *Zea mays*, *Sorghum bicolor*, *Setaria viridi*s and *Setaria italica*. Multiple sequence alignment of the BBX proteins was performed using MAFFT software, and the Maximum Likelihood (ML) phylogenetic tree was constructed with IQ-TREE (1,000 bootstrap replicates). Based on sequence similarity and the branching topology, these BBX proteins were grouped into five subfamilies (**Figure 4A**, **Supplementary Table 1**). This result is consistent with the phylogenetic classification previously observed in *Setaria italica*, *Setaria viridi*s and *Arabidopsis thaliana* (**Figure 1**).

We further quantified the number of *BBX* genes in each subfamily across all species. Notably, genes from subfamily V were entirely absent in Poaceae (monocots). Additionally, the number of *BBX* genes in subfamily II was significantly reduced in monocotyledons compared to the dicotyledonous species *Arabidopsis thaliana*. Among monocots, C_4_ grasses possessed fewer subfamily I genes than C_3_ grasses, while subfamily IV genes were relatively more abundant in C_4_ grasses. Interestingly, although *Sorghum bicolor* and *Zea mays* retained a small number of subfamily II genes, this subfamily was almost completely lost in *Setaria italica* and *Setaria viridis* (**Figures 4B, C**, **Supplementary Table 5**).

### 3.4 *BBXs* expression patterns and *cis*-acting element analysis

**Figure 5:**
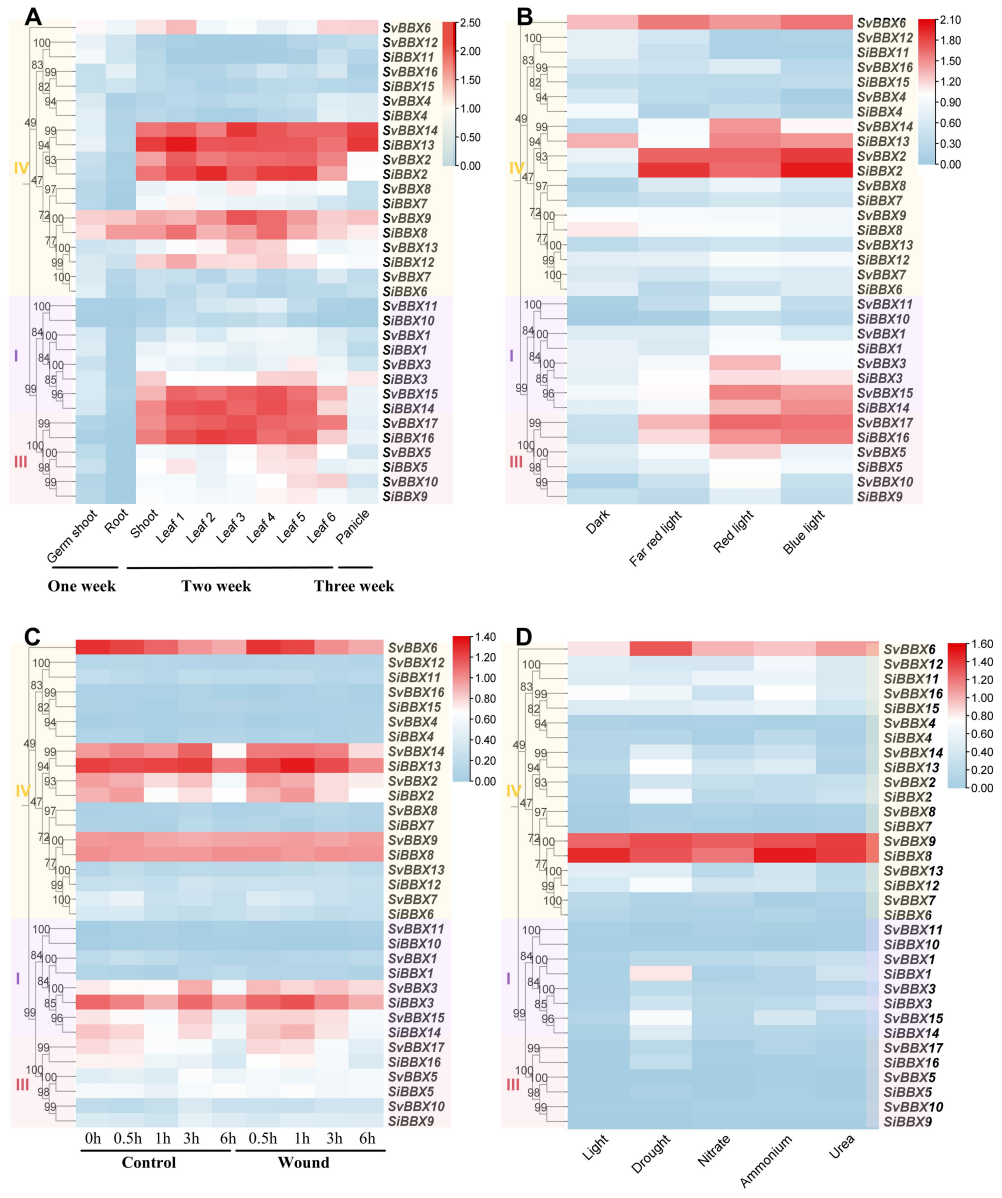
Expression profiles of *BBX* genes under different treatments and developmental stages in *Setaria*. **(A)** Expression profiles of *BBX* genes in Setaria italica at different developmental stages. **(B)** Expression of *BBX* genes in seedlings under light stress. **(C)** Expression profiles of *BBX* genes at different time points after wounding treatment. **(D)** Expression profiles of *BBX* genes in root tissues under various stress conditions. FPKM values were obtained from the Phytozome dataset and Log_10_(FPKM) for visualization. Heatmaps reflect qualitative expression patterns. Relative expression levels are indicated by the color scale on the right. *BBX* subfamily classification is shown on the left of each panel.

To further explore functional divergence within the *BBX* gene family, expression profiles were analyzed from publicly available RNA-seq datasets under different developmental stages and treatment conditions. We examined the expression of 16 *SiBBX* genes and 17 *SvBBXs* (**Figure 5**, **Supplementary Table 6**). The results showed that in *Setaria italica*, about 31% of the *SiBBXs* were highly expressed (FPKM > 30) in at least one developmental stage. For example, *SiBBX4* was highly expressed in panicles, while *SiBBX2, SiBBX3, SiBBX5, SiBBX9, SiBBX12, SiBBX13, SiBBX14*, and *SiBBX16* showed high expression in leaves. *SiBBX10* maintained low expression levels in all tissues. Similarly, in *Setaria viridis*, about 29% of *SvBBXs* were highly expressed in at least one tissue or developmental stage. In particular, *SvBBX2, SvBBX5, SvBBX9, SvBBX10, SvBBX13, SvBBX14, SvBBX15*, and *SvBBX17* showed strong expression in leaves and panicles. These results suggest that the *BBX* gene family in *Setaria* exhibits distinct expression patterns, which may indicate potential functional divergence across different tissues and stages (**Figure 5A**, **Supplementary Table 6**).

Under different light treatments, *BBX* genes showed a wide range of light-responsive expression. *SiBBX2, SiBBX13, SiBBX14, SiBBX16, SvBBX2, SvBBX6, SvBBX14, SvBBX15*, and *SvBBX17* were strongly induced by red and blue light. In contrast, *SiBBX8* was highly expressed under dark conditions, while *SiBBX9* and *SiBBX10* maintained low expression under all light treatments (**Figure 5B**, **Supplementary Table 6**).

Under wounding, the expression levels of *SiBBX1, SiBBX2, SiBBX3, SiBBX4*, and *SiBBX5* were significantly increased. In *Setaria viridis*, only *SvBBX5* and *SvBBX15* were clearly upregulated. Under light, drought, and nitrogen treatments, both *SiBBX8* and *SvBBX9* showed increased expression. These two genes exhibit expression patterns consistent with a potential role in nitrogen control, stress response, and light signaling (**Figures 5C, D**, **Supplementary Table 6**).

**Figure 6:**
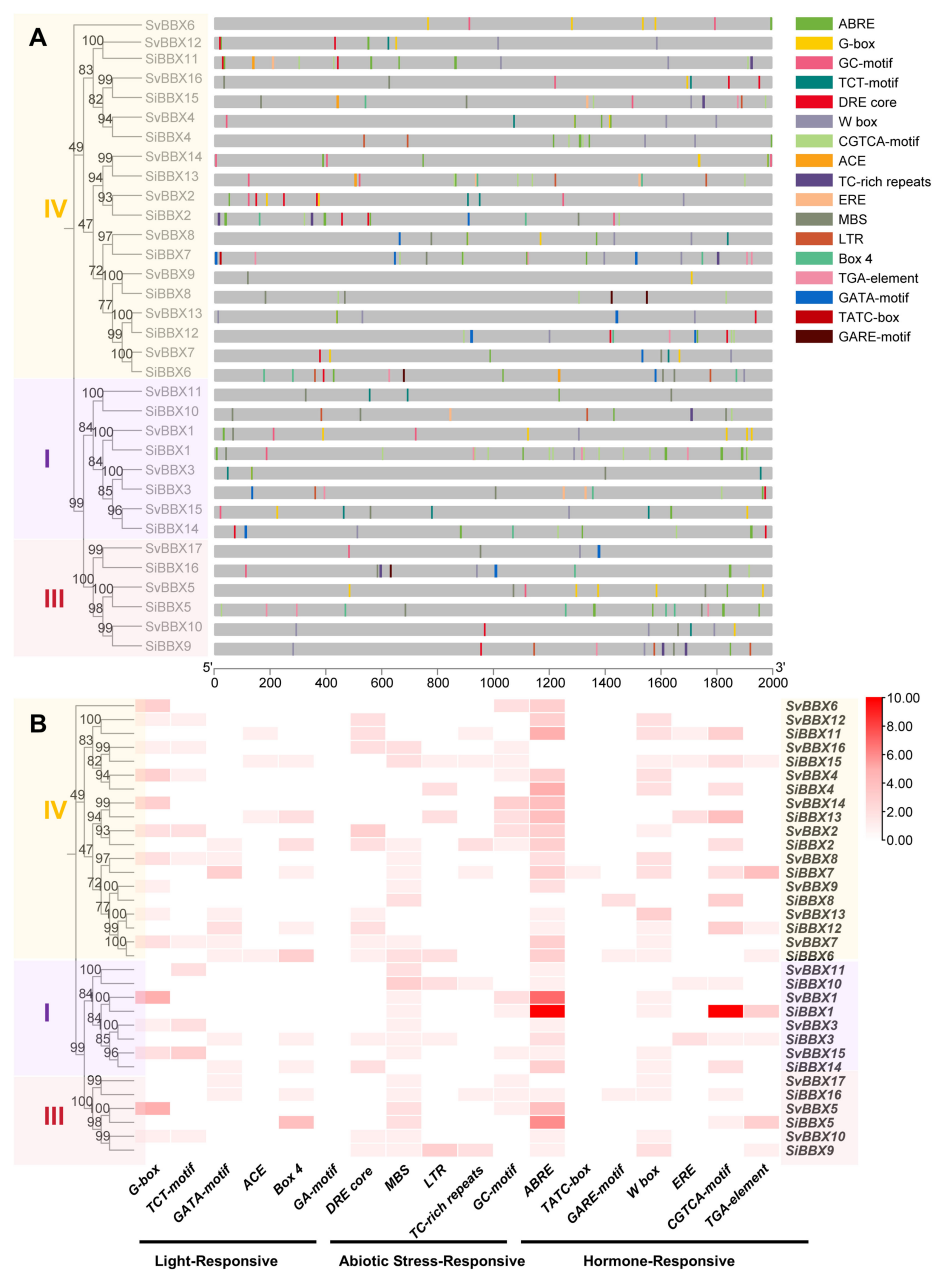
Analysis of cis-acting elements in the promoter region of *BBX* genes in *Setaria*. **(A)** Diagram of the *cis*-acting element locations in the 2000 bp promoter region upstream of the ATG of *BBX* genes in *Setaria*. **(B)** Statistical count of *cis*-acting elements responsive to different stress types in *Setaria*.

To investigate the regulation of *BBX* genes in *Setaria*, we examined the 2-kb promoter regions upstream of each gene to identify *cis*-acting elements (**Figure 6A**, **Supplementary Table 7**). These elements were grouped into three categories. The first group includes light-responsive elements such as *ACE* (involved in photoperiod response) and *Box 4* (enhances light-induced transcription). The second group includes elements related to abiotic stress, such as *DRE core* (recognized by *DREB/CBF* transcription factors) and *MBS* (*MYB* binding site for drought response). The third group includes hormone-responsive elements like *ABRE* (involved in ABA response), *TATC-box* (gibberellin response), and *W-box* (*WRKY* binding site involved in salicylic acid and defense responses).

To evaluate whether *cis*-acting elements were non-randomly enriched in *BBX* promoters, we performed Fisher’s exact enrichment tests using all genomic promoters as the background (**Supplementary Table 8**). The distribution of light-responsive elements were different between foxtail millet and green millet. *SiBBX* promoters showed a strong enrichment of *ACE* (FDR < 0.001, FE ≈ 127), whereas no enrichment was detected in *Setaria viridis*. In contrast, *G-box* elements were significantly depleted in *Setaria italica* (FDR < 1.7 × 10^-8^), suggesting a lineage-specific loss of this regulatory module during the evolutionary or domestication history of foxtail millet. Conversely, *SvBBX* promoters exhibited significant enrichment of the *TCT-motif* (FDR ≈ 0.021, FE ≈ 2.32), indicating preferential retention of a distinct light-signaling pathway in this species. *Box 4* also showed significant depletion in *Setaria viridis* (FDR < 1.1 × 10^-5^), further highlighting divergent trajectories in light-responsive promoter evolution between the two species (**Figure 6B**, **Supplementary Table 7, 8**).

Among the abiotic stress-related elements, *TC-rich* repeats were strongly enriched in *SiBBX* promoters (FDR < 5 × 10^-12^, FE ≈ 118), while no significant enrichment was detected in *Setaria viridis*. In contrast, low-temperature-responsive *LTR* elements were significantly depleted in *SvBBX* promoters (FDR ≈ 0.047), suggesting a possible reduction in cold-responsive regulatory capacity (**Figure 6B**, **Supplementary Table 7, 8**).

For hormone-responsive *cis*-elements, the jasmonate-responsive *CGTCA-motif* (FDR < 4 × 10^-8^) and the auxin-responsive *TGA-element* (FDR ≈ 0.047) were both significantly depleted in *Setaria viridis*, whereas these elements were retained in *Setaria italica*. These patterns indicate that the two species may have experienced distinct selective pressures shaping their hormone-mediated transcriptional networks (**Figure 6B**, **Supplementary Table 7, 8**).

### 3.5 Daily rhythmic analysis of SiBBXs under long-day photoperiod

**Figure 7:**
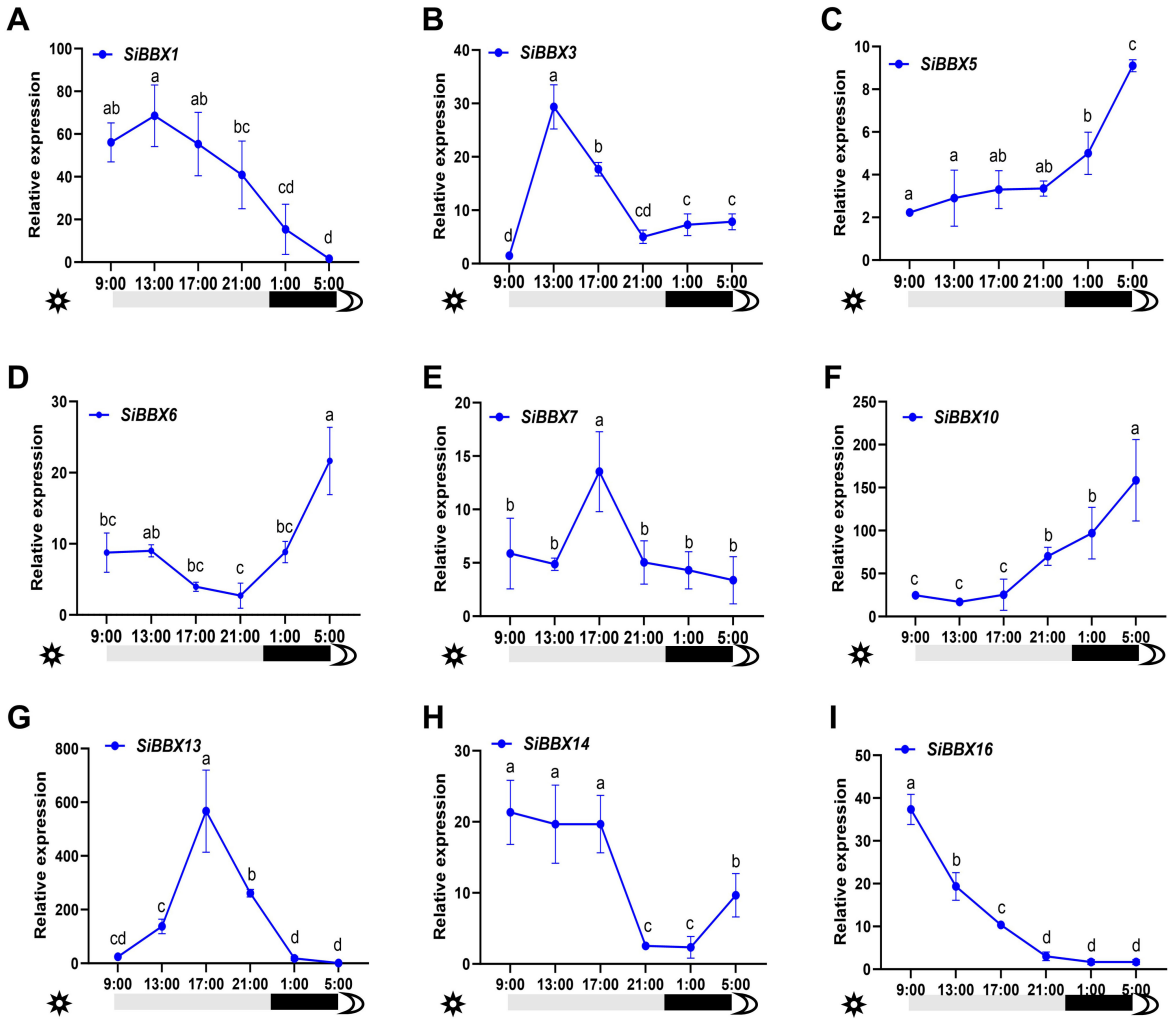
Expression patterns of SiBBX genes under long-day photoperiods. **(A–I)** Relative expression levels of nine foxtail millet genes, *SiBBX1*, *SiBBX3*, *SiBBX5*, *SiBBX6*, *SiBBX7*, *SiBBX10*, *SiBBX13*, *SiBBX14*, and *SiBBX16*, measured under long-day conditions (15 h light/9 h dark). Transcript levels were analyzed at six time points across a 24-h cycle (09:00, 13:00, 17:00, 21:00, 01:00, and 05:00) in the long-day cultivar Setaria italica ‘Longgu26’. Values represent means ± SD of three biological replicates. Different letters indicate significant differences (P < 0.05) as determined by one-way ANOVA followed by Tukey’s multiple comparisons test. Cullin was used as the internal reference gene, and relative expression levels were calculated using the 2^−ΔΔCt method, with *SiBBX1* expression at 05:00 normalized to 1.

To investigate whether *SiBBXs* are regulated by photoperiod in foxtail millet, we analyzed the transcriptional profiles of nine *SiBBXs* (*SiBBX1, SiBBX3, SiBBX5, SiBBX6, SiBBX7, SiBBX10, SiBBX13, SiBBX14*, and *SiBBX16*) in the “Longgu 26” under a long-day photoperiod (15 h light/9 h dark) at six time points (9:00, 13:00, 17:00, 21:00, 01:00, and 05:00) (**Figure 7**). *SiBBX1, SiBBX3, SiBBX13*, and *SiBBX14* maintained high transcript levels during the light period (9:00–24:00) and decreased rapidly after entering the dark period. *SiBBX1* and *SiBBX3* peaked at midday (13:00) and then declined steadily, remaining low during the dark period. *SiBBX7* and *SiBBX13* showed peak in the light period (17:00), followed by a significant decrease in dark period. *SiBBX14* and *SiBBX16* peaked during the light period (9:00), gradually declined afterward, and showed a slight increase again during the dark period (05:00). In contrast, *SiBBX5* and *SiBBX10* exhibited clear dark-induced expression patterns, with transcript levels increasing steadily during the dark period (00:00–09:00), reaching their highest levels at the dark period (5:00) and showing higher expression than during the light period. Additionally, *SiBBX6* displayed a significant decrease in the light period (21:00) and then increasing during the dark period. According to these results, *SiBBXs* may play distinct roles in photoperiod signaling.

### 3.6 Subcellular localization analysis of *SiBBXs*

**Figure 8:**
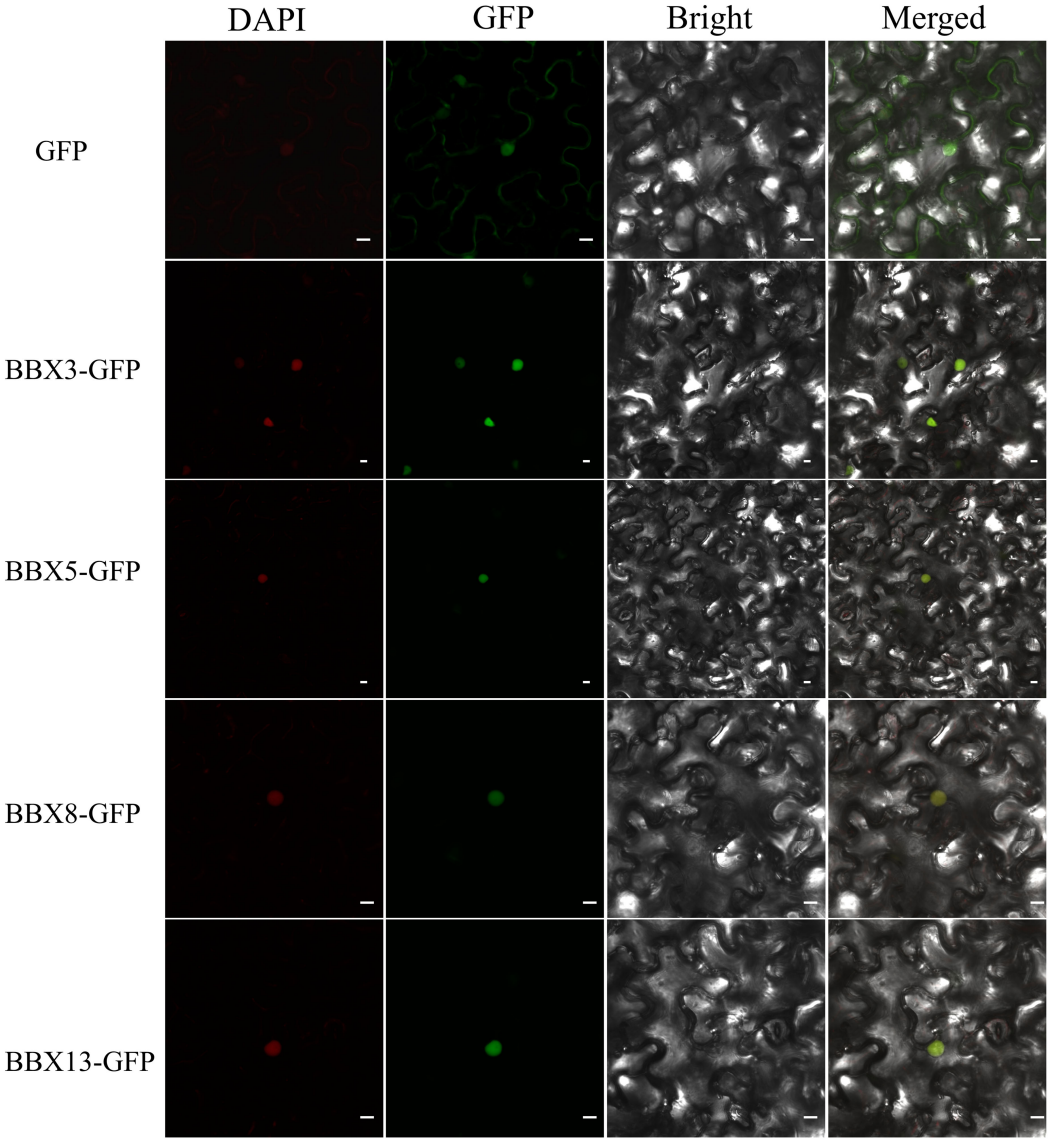
Analysis of the subcellular localization of *SiBBXs* in *Nicotiana benthamiana* leaves. GFP, GFP channel; DAPI was used as stain for cell nucleus. Bars = 20 μm.

The subcellular localization of proteins can provide insights into their protein functions. Since *SiBBXs* exhibit significant daily rhythmic expression changes under long-day photoperiod, we further analyzed the subcellular localization of some *SiBBXs*. Heterologous expression results in tobacco leaves showed that *SiBBX3*, *SiBBX5*, *SiBBX8*, and *SiBBX13* are primarily localized within the cell nucleus (**Figure 8**). These results suggested that *SiBBX3*, *SiBBX5*, *SiBBX8*, and *SiBBX13* may function as transcriptional regulators in the light response, consistent with their predicted roles as transcription factors. However, localization patterns of the remaining family members remain to be validated in future studies.

## 4 Discussion

The transcription factors of the *B-box* (*BBX*) gene family are essential for controlling photoperiod, flowering timing, and plant tolerance to environmental stress (Khanna et al., 2009; Gangappa and Botto, 2014). Additionally, The *BBX* in plant evolution is receiving increasing attention. Earlier research has identified 19 *BBX* genes in foxtail millet and reported genome-wide characterization of *BBX* genes in several grasses, including maize, rice, sorghum, and stiff brome, with *OsBBXs* shown to participate in abiotic stress responses (Shalmani et al., 2019). However, the molecular evolutionary mechanisms of *BBX* genes in grass species, and their roles in light responses remain unclear. This study systematically identified and analyzed the *BBX* gene family in two typical C_4_ plants of the *Setaria*, foxtail millet and green millet using a dual screening strategy involving Hidden Markov Model (HMM) domain search and BLASTP. However, 16 and 17 *BBX* genes were identified in foxtail millet and green millet by genome-wide analysis, respectively (**Figure 2**, **Supplementary Table 1**). Compared to the number of genes reported in previous studies of grass crops (maize, rice, sorghum, foxtail millet), the results of this study are relatively fewer, mainly due to the dual screening strategy and lower e-value thresholds (Shalmani et al., 2019).

### 4.1 Evolutionary dynamics of *BBX* gene family in *Setaria*

In biological evolution, segmental and tandem duplication jointly promote the formation of gene family (Cannon et al., 2004; Hofberger et al., 2015). In this study, we identified five pairs of duplicate genes involving ten genes in foxtail millet and green millet (**Figure 3B**, **Supplementary Table 2**). Duplication type analysis showed that these duplicate genes mainly originated from dispersed duplication (**Figure 3C**, **Supplementary Table 3**). Previous studies have shown that young dispersed duplicate gene pairs in *Arabidopsis thaliana* exhibit significant asymmetric expression and accelerating functional differentiation between the two genes (Owens et al., 2013). Similarly, in grasses, this small-scale duplication (SSD) has also been found to often help genes quickly acquire new functions (Jiang and Assis, 2019). Therefore, we speculate that this dispersed duplication in foxtail millet and green millet may have driven the differentiation of gene functions and the acquisition of new functions, especially in the regulation of responses to different photoperiods and environmental stresses. Interestingly, all duplicated *BBX* genes in foxtail millet and green millet have undergone strong purifying selection (Ka/Ks < 1), suggesting that these genes are highly conserved evolutionarily and may perform highly conserved core biological functions (**Supplementary Table 2**). The conserved motifs and gene structures also indicate the conservation of these genes (**Supplementary Figure 1**). The dispersed duplication initially drives functional innovation, but the resulting duplicates are subsequently stabilized by purifying selection to preserve core biological functions. We speculate that dispersed duplication may have promoted the diversification of gene functions, with some genes being screened by selective pressure and evolving into core genes in *Setaria*.

Synteny analysis helps track how gene duplication affects the evolution of gene family (Flagel and Wendel, 2009). Our results show that foxtail millet shares extensive synteny with the other five grass species, reflecting a largely conserved genomic context within Poaceae. Nearly all *SiBBX* genes are retained in conserved syntenic blocks across *Oryza sativa*, *Triticum aestivum*, *Zea mays*, *Sorghum bicolor*, and *Setaria viridis*, except for *SiBBX7*, which is syntenically conserved only in *Setaria viridis*, *Setaria bicolor*, and *Oryza sativa* (**Figures 4A-C**; **Supplementary Table 2**). These *SiBBXs* (excep *SiBBX7*) possess multiple high-confidence orthologous syntenic pairs in all five grasses, indicating inheritance from ancient genomic regions formed during the grass-common whole-genome duplication (WGD) ~70 MYA (Paterson et al., 2004; Wang et al., 2005, 2009). These conserved BBX orthologs exhibit strong purifying selection (Ka/Ks < 1) and divergence times of < 50 MYA (**Figures 4A-C**, **Supplementary Table 2**), supporting their evolutionary stability and functional importance. In addition to these ancient duplications, *Setaria* also retains a set of much younger syntenic pairs. Several *Setaria italica*-*Setaria viridis* orthologs (e.g., *SiBBX1-SvBBX1*, *SiBBX6-SvBBX7*, *SiBBX7-SvBBX8*, *SiBBX10-SvBBX11*, *SiBBX11-SvBBX12*, *SiBBX13-SvBBX14* and *SiBBX16-SvBBX17*) show extremely low Ks values (< 0.02) and estimated divergence times < 1 MYA (**Figures 4A-C**, **Supplementary Table 2**), consistent with Panicoideae-specific small-scale duplications rather than ancient genome-wide events. This indicates that multiple layers of duplication (ancient WGD plus recent SSD events) jointly shaped *BBX* gene retention and diversification within Setaria. Notably, the two genes *SiBBX1* and *SiBBX11* are present in all six species and are even associated with two or more syngeneic gene pairs (**Figures 4A-C**, **Supplementary Table 2**). *SiBBX7* is only associated with syntenic gene pairs in *Setaria viridis*, *Sorghum bicolor*, and *Oryza sativa*.(**Figures 4A-C**, **Supplementary Table 2**). Although *SiBBX1*, *SiBBX7* and *SiBBX11* show very low abundance in the RNA-seq dataset (**Figure 1A**, **Supplementary Table 6**), their preservation in deeply conserved syntenic regions across all analyzed species strongly suggests that they are maintained under long-term evolutionary constraints. This indicates that low transcript abundance does not necessarily imply functional insignificance, as these genes may respond to specific developmental stages or environmental cues not represented in the public dataset. Moreover, our qRT-PCR assays demonstrated clear daily expression for *SiBBX1* and *SiBBX7* with low baseline RNA-seq abundance (**Figure 7**), further supporting that their transcription is condition-dependent rather than universally silent.

Based on molecular clock analysis using the synaptonemal replacement rate (6.5 × 10^-9^), we found that the divergence time of orthologous genes between foxtail millet and rice/wheat was approximately 26.9 million years ago (MYA), approximately 19.2 MYA for maize/sorghum, and only approximately 0.8 MYA for green millet (**Figures 4D, E**, **Supplementary Table 2**). Previous studies have shown that the Panicoideae diverged from the Oryzoideae around 55 MYA, and that the tribe Panicoideae and the tribe Sorghumideae diverged around 33 MYA, and the divergence time between foxtail millet and green millet was approximately 1 MYA (Zhang et al., 2012; Ma et al., 2021), which is consistent with our findings (**Supplementary Figure 2**). This also indicates that the *BBX* gene family has undergone purifying selection, maintaining high sequence conservation even after subfamily and tribe divergence, suggesting its functional importance for biological adaptation. This provides important clues for understanding the functional evolution of light regulated genes in the phylogeny and domestication of Poaceae.

Plants adapt to local environments by selecting the optimal phenotypes through natural or artificial selection (Hou et al., 2025). Notably, *BBX* gene family gain and loss analysis suggests adaptive reduction during the evolution of C4 grasses (**Figure 5**, **Supplementary Figure 2**, **Supplementary Table 4**). However, the number of *BBX* genes in maize has slightly increased, which may be associated with maize’s whole-genome duplication and increased demand for light regulation during C_4_ evolution (Maere et al., 2005; Hoff, 2010; Yu et al., 2015). We speculate that continuous gene loss may improve the light response efficiency of C_4_ grasses by removing regulatory redundancy. Although this pattern may partially reflect adaptive pressures associated with C_4_ photosynthesis or photoperiod-related ecological niches, several non-adaptive evolutionary mechanisms could equally account for *BBX* gene reduction (Chung et al., 2023; Lynch, 2007). The evolutionary pattern of foxtail millet is a typical example. The foxtail millet genome has a transposon content of up to ~46%, which can directly induce gene variation through insertion or excision (He et al., 2023). Such transposon-mediated insertions or excisions are inferred to provide raw materials for BBX gene variation. On this basis, the foxtail millet pan-genome ultimately retains approximately 29.4% dispensable genes under relaxed selection pressure (He et al., 2023). Notably, the presence of these dispensable genes may allow non-adaptively driven BBX gene gain or loss without compromising foxtail millet’s survival, thereby providing a genetic background for the subsequent selective retention of beneficial BBX variants. Meanwhile, the *BBX* gene duplication patterns in foxtail millet are specific, the frequencies of dispersed, proximal, and tandem are all higher than the genome-wide average (**Figure 3C**), this feature is inferred to create more possibilities for non-adaptively driven *BBX* gene gain/loss. Furthermore, syntenic *SiBBX* gene pairs in foxtail millet are mainly under purifying selection (**Supplementary Table 2**). This selection pattern maintains gene functional conservation and is also inferred to potentially lead to functional overlap among multi-copy *BBX* genes. In addition, genome size variation and annotation differences could contribute to observed *BBX* gene number differences. For instance, wheat harbors a notably large *BBX* gene family, with 96 identified members. This is primarily attributed to wheat being an allohexaploid, which has a relatively large genome size. (Cheng et al., 2024). Importantly, all seven genomes analyzed in this study are high-quality, well-annotated reference assemblies from Phytozome. Although we cannot completely rule out the possibility that variations in genome quality and annotation completeness may cause mistakes in the detection of BBX gene gain or loss, we propose that their impact is relatively minor.

The divergence time estimated in this study is based on the average synonymous substitution rate, which may overlook potential rate heterogeneity among different gene loci. In addition, phylogenetic analysis revealed that the *BBX* genes of the dicotyledonous plant (*Arabidopsis thaliana*) are divided into five subfamilies, while the *BBX* genes of monocotyledonous grasses, including C_4_ plants (sorghum and maize) and C_3_ plants (rice and wheat), mainly belong to four subfamilies: I, II, III, and IV. Interestingly, we observed a significant absence of the subfamily II in the *Setaria* (foxtail millet and green millet), while subfamily IV genes uniquely expanded (**Figure 5**, **Supplementary Table 5**). This asymmetric pattern of loss and gain may reflect the pressures experienced during the differentiation process of different tribes within the Poaceae, potentially linked to the evolution of C_4_ photosynthesis and photoperiod adaptation.

### 4.2 Functional diversification in light and stress responses

In multiple species, the *BBX* gene are involved in various biological processes such as light-induced flowering, photomorphogenesis, and responses to abiotic stress (Gangappa and Botto, 2014; Bursch et al., 2020; Liu et al., 2022; Yang et al., 2024). *SiBBX2* and *SiBBX13* belong to subfamily IV, which were significantly upregulated under red and blue light conditions, and contained more light-responsive *cis*-acting elements (**Figures 1**, **6**, **Supplementary Tables 6–8**). Subfamily IV usually contains more light-responsive *cis*-acting elements (Sarmiento, 2013), which is consistent with our results (**Figure 6**, **Supplementary Table 7, 8**). Similar studies in *Oryza sativa* and *Arabidopsis thaliana* have also shown that subfamily IV *BBX* genes act as regulators of light signals and hormone pathways, regulating photomorphogenesis and flowering (Bai et al., 2016; Ding et al., 2018; Yang et al., 2024). Notably, *SiBBX2*, *SiBBX13*, and *AtBBX24* are homologous genes. Previous studies have showed that *AtBBX24* regulates light sensitivity and salt stress tolerance (Xie et al., 2024). Therefore, we speculate *SiBBX2* and *SiBBX13* may be involved in light response. In addition, some *BBX* genes in foxtail millet and green millet were induced under abiotic stress (**Figure 1**, **Supplementary Table 6**). At the same time, we found that some *BBX* genes contain many *ABRE*, which is ABA-related *cis*-acting element binding sites (**Figure 6**, **Supplementary Table 7**). This supports earlier reports that *BBX* genes may play a role in drought response, ABA signal transduction, and other abiotic stress responses (Xu et al., 2020; Bandara et al., 2022; Wu et al., 2023). Although there are differences in the number of genes among different species, the *BBX* gene is conserved in terms of light signaling and stress adaptation. Additionally, our results revealed that although the *BBX* gene family in foxtail millet and green millet exhibits high sequence homology. However, there are significant differences in their expression patterns and *cis*-acting element (**Figures 1**, **6**, **Supplementary Tables 6-8**). This suggests that the *BBX* gene family has undergone species-specific *cis*-acting element remodeling in Setaria, which may have contributed to potential functional differentiation. For example, the light-responsive elements in foxtail millet are mainly *ACE*, while those in green millet are mainly *TCT*-motif, which may lead to differences in light response (**Supplementary Table 8**). Analyzing abiotic stress-responsive *cis*-acting elements, we found that foxtail millet has more *LTR* and *TC* elements (**Supplementary Table 8**), which is associated with cold and defense responses (Parajuli et al., 2024), while green millet lacks these elements. In hormone-responsive *cis*-acting elements, green millet lacks elements related to auxin (*TGA-element*) and jasmonic acid (*CGTCA-motif*), which might cause differences in hormone regulation pathways compared to foxtail millet (**Supplementary Table 8**).

### 4.3 Daily rhythmic expression in long-day photoperiod and transcriptional control

The expression analysis of daily rhythms in long-day photoperiod indicates that the circadian clock and photoperiod may regulate the *SiBBXs*. Under a long-day photoperiod, *SiBBX1* and *SiBBX3* show rhythmic expression patterns, peaking at 13:00 (**Figure 7**). Both genes belong to the I subfamily and are homologous to *AtCO* (*AtBBX1*) (**Figure 2**). *SiBBX3* is localized in the cell nucleus (**Figure 8**). In *Arabidopsis thaliana* and *Oryza sativa*, BBX proteins (CO, Hd1), which is thought to be a nuclear localization signal, can regulate flowering time through circadian rhythm and photoperiodic pathways (Cheng and Wang, 2005; Hassidim et al., 2009; Valverde, 2011; Xu et al., 2022; Yang et al., 2024). These findings imply that *SiBBX1* and *SiBBX3* in foxtail millet likely perform similar functional roles. *SiBBX5* and *SiBBX16* have opposite expression patterns and belong to the III subfamily (**Figures 2**, **5**, **7**, **Supplementary Table 1**). *SiBBX5* is localized in the cell nucleus (**Figure 8**). The two proteins are homologous to BBX14/15 in *Arabidopsis thaliana*, which are involved in the light response, seedling development, and abiotic stress, suggesting a potentially similar role for SiBBX5 and SiBBX16 (Soitamo et al., 2008; Atanasov et al., 2023; Buelbuel et al., 2023). *SiBBX7* and *SiBBX13* have similar expression patterns (**Figure 7**), both belonging to the IV subfamily (**Figures 2**, **5**, **Supplementary Table 1**), and show homology with the *AtBBX23/24/25*. The location of SiBBX13 is in the cell nucleus (**Figure 8**). Previous researches have indicated that *AtBBX23/24/25* are light-dependent and associated with morphogenesis (Indorf et al., 2007; Sentandreu et al., 2011; Gangappa et al., 2013; Sarmiento, 2013; Xie et al., 2024).

The identification of *BBX* genes with photoperiod-responsive expression patterns may provide potential molecular breeding targets for improving the heading date and environmental adaptability of crops. In the future, transgenic technologies such as genome editing should be used to perform functional analyses of candidate *BBX* genes to elucidate their regulatory networks and downstream targets, which will enhance our comprehension of the photoperiod adaptation mechanisms of C_4_ crops.

This study systematically investigated the evolutionary dynamics and functional diversification of the *BBX* gene family in C_4_ grasses, using *Setaria italica* and *Setaria viridis* as model systems. However, the current work has several limitations, our conclusions rely heavily on bioinformatic predictions and comparative genomic analyses, with a notable lack of direct experimental validation. To address this gap in future research, we intend to generate CRISPR/Cas9-mediated knockout and overexpression lines to validate the biological functions of candidate *BBX* genes, specifically their roles in photoperiod regulation, stress tolerance, and flowering time control, and will further perform genetic complementation experiments. These approaches are expected to establish causal relationships between the key characteristics of *BBX* genes and their proposed functions, thereby further enhancing the reliability of our conclusions.

## 5 Conclusion

In conclusion, this study systematically elucidated the evolutionary history and functional diversification of the *BBX* transcription factor family in *Setaria viridi*s and *Setaria italica*. We revealed that the *BBX* gene family contraction and modular functional differentiation are consistent with a potential synergistic role in photoperiodic adaptation in C_4_ grasses. The identified core *BBX* genes and their stress- and light-responsive regulatory elements provide valuable potential molecular targets for improving heading and stress tolerance in cereal crops. Future studies should emphasize functional validation of candidate *BBX* genes and their regulatory networks, which will support breeding strategies for environmental adaptation in C_4_ crops.

## Data Availability

The original contributions presented in the study are included in the article/**Supplementary Material**. Further inquiries can be directed to the corresponding authors.
