# Supplementary figures and images for: Evolutionary dynamics and functional diversification of BBX transcription factors in C4 grasses from Setaria italica and Setaria viridis
Source: Front Plant Sci. 2026 Jan 22;16:1701242. doi: 10.3389/fpls.2025.1701242 (PMC12872835; doi:10.3389/fpls.2025.1701242)

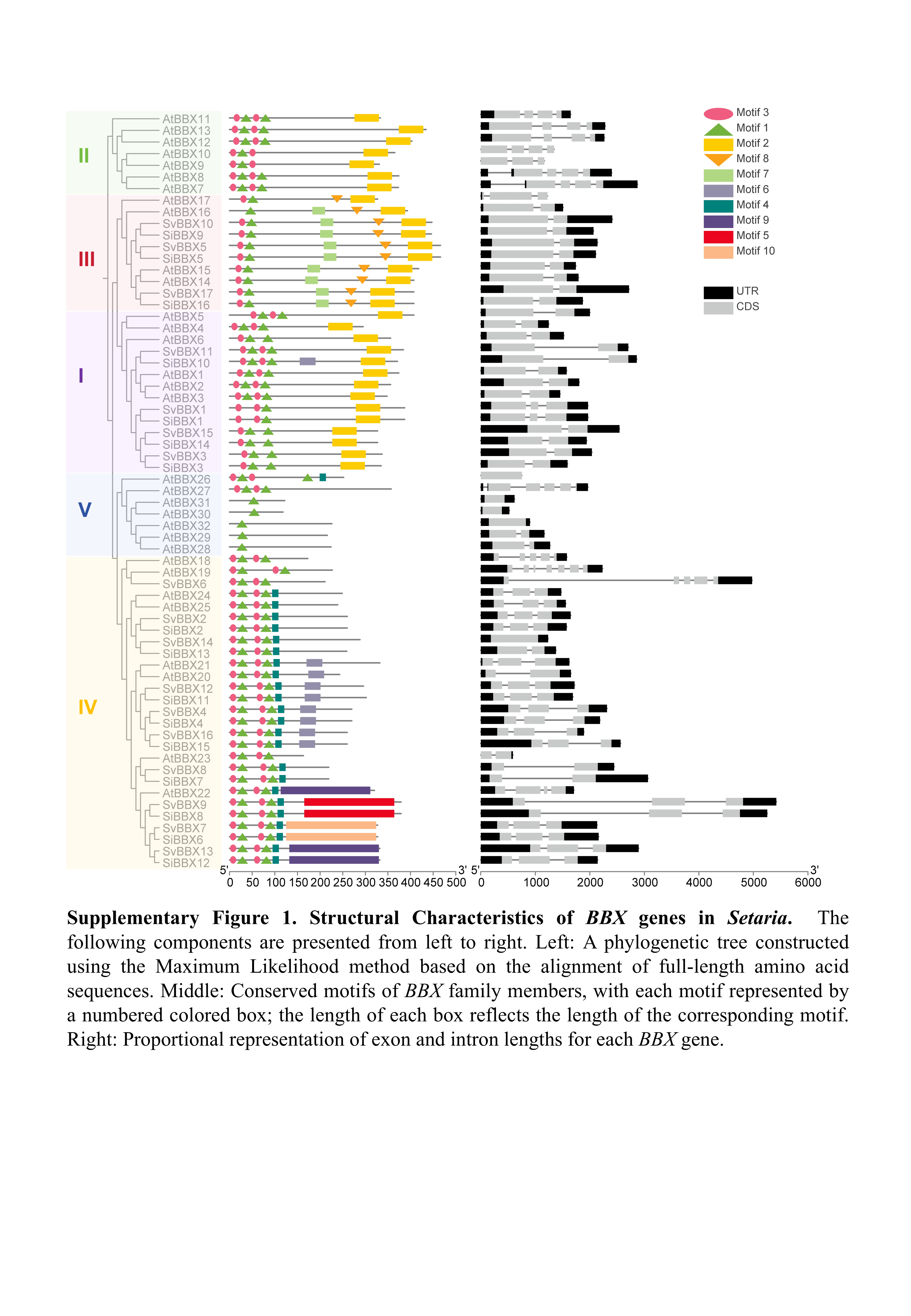

Supplement: Supplementary file 1 [file Image1.tif]

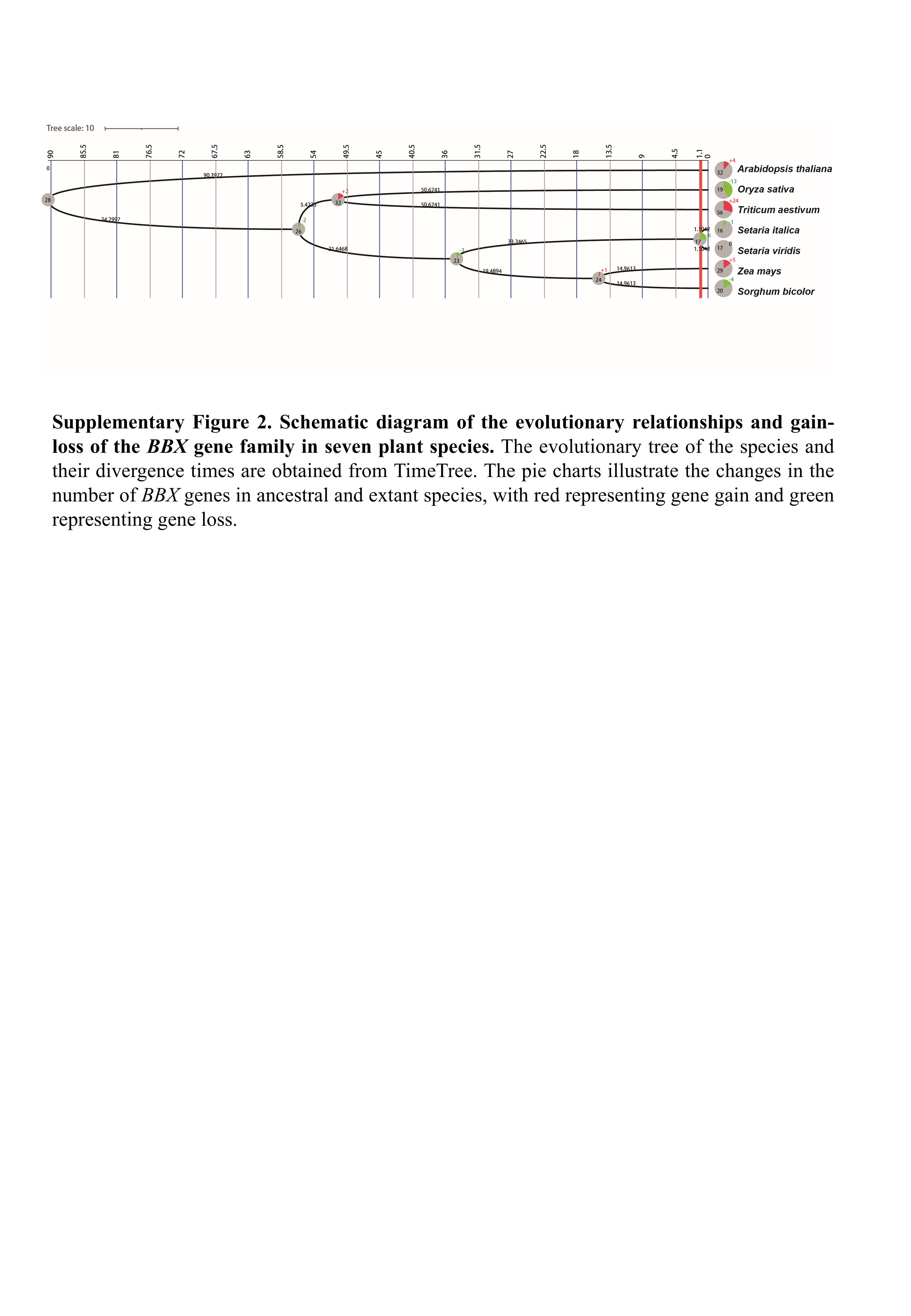

Supplement: Supplementary file 2 [file Image2.tif]
